# The relationship between depression and overall, general psychopathology, positive, and negative symptoms in people with schizophrenia spectrum disorders: a cross-sectional study

**DOI:** 10.1192/j.eurpsy.2024.797

**Published:** 2024-08-27

**Authors:** F. Bartoli, A. Calabrese, F. Moretti, M. Castiglioni, L. Prestifilippo, A. De Pietra, M. Gazzola, P. Camera, C. Crocamo, G. Carrà

**Affiliations:** ^1^Department of Medicine and Surgery, University of Milano-Bicocca , Monza; ^2^Department of Mental Health and Addiction Services, ASST Nord Milano, Sesto San Giovanni, Milan, Italy; ^3^Division of Psychiatry, University College London, London, United Kingdom

## Abstract

**Introduction:**

Depressive symptoms are a common occurrence in people suffering from schizophrenia spectrum disorders (SSDs), representing a separate domain that interacts in peculiar ways with positive and negative symptoms. Nonetheless, available evidence on the relationship between depression and key clinical dimensions of SSDs is limited.

**Objectives:**

To increase the knowledge regarding depression in SSDs, we performed a cross-sectional study aimed to investigate the association of depressive symptoms with overall, general psychopathology, positive, and negative symptoms in individuals with SSDs.

**Methods:**

Adult people with SSDs were recruited from two psychiatric inpatient units in the northern area of the Metropolitan City of Milan from May 2020 to March 2023. Study participants with a Calgary Depression Scale for Schizophrenia score >6 were rated as depressed. Symptom severity was assessed by using the Positive and Negative Syndrome Scale (PANSS). Variables associated with depression at the univariate level were included into two multiple logistic regression models to analyse the association between depression and PANSS overall score as well as General Psychopathology, Positive, and Negative sub-scores.

**Results:**

A total of 231 subjects with SSDs were included. Among them, approximately one third (N=78; 33.8%) reported depressive symptoms. Multiple logistic regression models suggested that depression in individiuals with SSDs was associated with higher overall (p<0.001) and General Psychopathology (p<0.001) PANSS scores. Conversely, an inverse relationship between depression and positive symptoms was found (p=0.002). Negative symptoms were not associated with depression (p=0.210).

**Conclusions:**

Our findings suggest that people affected by comorbid SSDs and depression have more severe overall and General Psychopathology symptoms according to PANSS scores, as well as lower levels of positive symptoms. Further investigations are needed to evaluate the generalisability of these findings and to improve the clinical management of people with SSDs and depression.

**Disclosure of Interest:**

None Declared

